# Pharmacokinetics and Excretion Studies on CDRI-85/92, an Antiulcer Proton Pump Inhibitor

**DOI:** 10.3797/scipharm.1111-05

**Published:** 2011-12-12

**Authors:** Pratima Srivastava

**Affiliations:** Pharmacokinetics and Drug Metabolism, Central Drug Research Institute, Lucknow, India. E-mail: pratimacdri@rediffmail.com

**Keywords:** CDRI Compound 85/92, Antiulcer pharmacophore, Bioavailability, Pharmacokinetics, Excretion, PPI

## Abstract

CDRI 85/92 is an antiulcer pharmacophore and a proton pump inhibitor, which is in an advanced stage of preclinical trials. In view of its importance, pharmacokinetic and excretion were studied in *Sprague Dawley* rats after administering 20 mg/kg oral and intravenous doses. The compound was detectable in the serum samples as early as 5 min post-oral administration. The compound was eliminated slowly from serum with an elimination half-life of 2.1 h. Following the 20 mg/kg oral dose, maximum serum concentration (C_max_) was found to be 469.28 ± 45.52 ng/ml after 1.0 h. Based on AUC values, the absolute bioavailability of the CDRI 85/92 was 70.5% after oral administration. It was found to be excreted in urine (~15% of the dose) in intravenously treated (bile duct cannulated as well as noncannulated) rats, whereas bile and feces depicted insignificant levels of the compound.

## Introduction

**Fig. 1:**
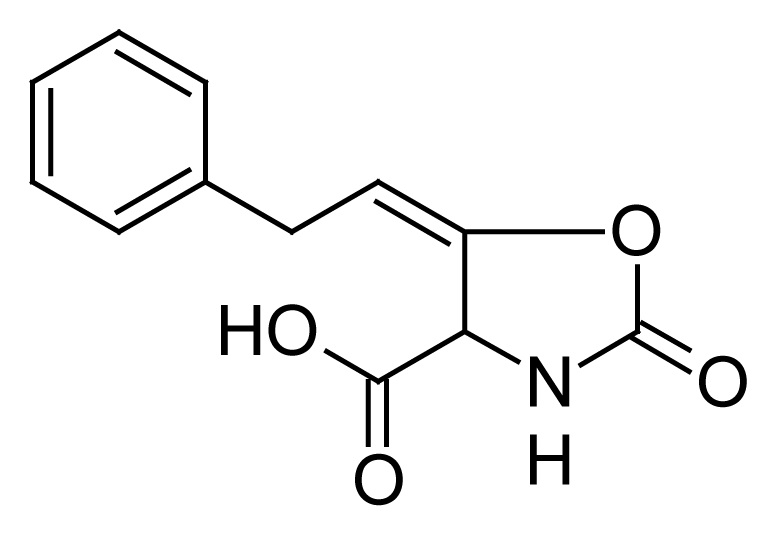
Chemical structure of CDRI-85/92

Proton pumps (PPs), specific for H^+^, K^+^-ATPase, are responsible for the terminal step in the gastric acid secretion. Gastric acid secretion is significantly increased in multiple diseases and can lead to the formation of gastric ulcers [1]. Effective antiulcer pharmacophores and/or proton pump inhibitors are considered to be most promising in terms of providing acid suppression, pain relief and ulcer healing properties. At present, proton pump inhibitors (PPIs) remain the most effective available therapy. Compound CDRI-85/92, (5*E*)-2-oxo-5-(2-phenylethylidene)-1,3-oxazolidine-4-carboxylic acid (Fig. 1), developed by Central Drug Research Institute, in Lucknow, India, is an antiulcer pharmacophore. It possesses potent proton pump inhibition profile, providing a selective and competitive inhibition of the gastric proton pump and not inhibiting the proton pumps of the kidney and bone. Omeprazole (PPI), a drug of choice for treatment of antiulcer, is an irreversible proton pump inhibitor and decreases the inhibition % of the acidity by increasing the concentration of ATP [2]. The % inhibition in the free acidity of CDRI-85/92 fairly compares with that of Omeprazole. Compound CDRI-85/92 has also shown good cytoprotective activity [3]. It possesses appreciable physicochemical properties {mol wt 233; aqueous solubility >500μg/ml; log P 0.5; 2 pK_a_ 3.65 and 11.98; Ka (absorption rate constant) 0.12 /h was alike at acidic and basic pHs, indicative of the presence of acidic and basic groups in the molecule; protein binding ~ 35%}. The compound is currently in an advanced stage of preclinical trials. However, no full insight of the pharmacokinetic and excretion data is available.

To develop it as a candidate drug, the aim of this study was to determine the complete Pharmacokinetics and Excretion profile of CDRI-85/92 in bile, feces and urine, after 20 mg/kg oral and intravenous doses in male *Sprague Dawley* rats.

## Experimental

### Chemicals and solvents

Compound CDRI-85/92 was synthesised in house (purity >99%) and was used in the present study. HPLC grade acetonitrile was purchased from Ranbaxy Laboratories, (SAS Nagar, India). Analytical grade glacial acetic acid was procured from Qualigens Fine Chemicals, (Bombay, India). Ammonium acetate was obtained from HIPersolv, (England, UK). Triple distilled water from all Quartz glass apparatus was used in the preparation of the buffer. Blood was collected from healthy male *Sprague Dawley* rats and was centrifuged to separate serum so as to generate a drug-free serum pool.

### Preparation of formulations

Solution formulations (10 and 20 mg/ml) of CDRI-85/92 were prepared by dissolving 200 and 400 mg in 20 ml of triple distilled water (isotonic solution: compound was completely soluble at the given strength) for oral and intravenous dosing.

### Animal study

Adult healthy male *Sprague Dawley* rats weighing 250 ± 25 g were obtained from Laboratory Animal Division of the Central Drug Research Institute (Lucknow, India) and were maintained under standard in house conditions. They were kept in plastic cages and were allowed free access to standard pellet diet (Lipton India Ltd., Bangalore) and tap water *ad libitum*. All experiments in rats, euthanasia and disposal of carcasses were carried out as per the guidelines laid down by the local ethics committee for animal experimentation. Care was taken to minimize the trauma as a result of pain during all the surgical procedures and blood sampling by the use of ether anesthesia. The rats were maintained on a 12 h light-dark cycle for at least one week before use.

### Instrumentation and chromatographic conditions

The HPLC system consisted of a solvent delivery system with a controller (Kontron HPLC System, Unicam, Cambridge, UK) equipped with a 7125 injector (Rheodyne, Berkely, USA) fitted with a fixed 20-μl loop and a Kontron UV spectrophotometer detector (Uvikon 730S LC set at 250 nm). The samples were injected with a 100 μl syringe. Separation was achieved on a C-18 column (5 μm, 220 × 4.6 mm, id), coupled with a guard column packed with the same material (5 μm, 30 × 4.6 mm, id), (E-Merck, Darmstadt FR Germany; No. 619429). The chromatograms were integrated using CR1B Chromatopac integrator (Shimadzu, Kyoto, Japan). The HPLC system was equilibrated for approximately 30 min at a flow rate of 1 ml/min before analysis commenced. A vortex-mixer (Thermolyne, India), ultrasonic bath (Bransonic, Shelto, CY), a Model SVC-220H Speed vac concentrator (Savant, NY) or Heto/Maxi Dry Plus, Germany) and a Model C-30 centrifuge (Remi, India) were used for sample preparations. The mobile phase was prepared by mixing methanol, acetonitrile and 10 mM ammonium acetate buffer (pH 4.0) (filtered through 0.22 μm Millipore filter) (29:1:70, v/v/v). The mobile phase was degassed for 15 min in the sonicator before use and was pumped at a flow rate of 1 ml/min. The chromatography was performed at ambient temperature.

### Pharmacokinetics of CDRI-85/92

As the first step of experiment, an aqueous solution formulation of the compound was administered intravenously to conscious rats via the caudal vein at a dose of 20 mg/kg in a volume of approximately 1.0 ml/kg/rat. Before the experiment, the tail was cleaned with luke warm water and the caudal vein was dilated using xylene. Blood (approx. 1.2 ml) was collected by cardiac puncture under light ether anesthesia at 0.08, 0.25, 0.5, 1, 2, 3, 4, 5, 6, 8, 12, 24 and 48 h post dose. In the second study, conscious rats, fasted overnight, were fed orally at a dose of 20 mg/kg in a volume of 2.0 ml/kg/rat. After drug delivery, blood was collected at the same time points used in the intravenous study. Three rats were utilized for each time point and not more than three or four (later in case of 48 hr) blood samples from each rat, two of which were by intracardiac during the absorption-distribution phase (till 5 hr) and one from the venecava in the terminal phase (6 hr onwards), was withdrawn. Together in two sets of studies, there were 24 rats. (Pl refer to Sampling schedule: Table 1). The total volume of the blood withdrawn within 24 h from cardiac puncture was less than 10% of the total blood volume. Blood was allowed to clot in sealed glass tubes and centrifuged at 2000 rpm. The serum was separated and stored at −60°C until analyses.

### Urinary, fecal and biliary excretion of CDRI-85/92

Urinary, fecal and biliary excretion of CDRI-85/92 was studied in rats (n=3 per group) after a single 20 mg/kg oral and intravenous dose. Following treatment with CDRI-85/92, urine and feces samples were collected during 24 h with 12 h intervals. Urine was measured and kept in parafilm sealed tubes at −60°C till analyses. Feces samples were dried at 37°C, weighed, ground to fine powder using mortar-pestle and stored in desiccator until analyses. Untreated rats were housed in metabolic cages with food and water *ad libitum* and their feces and urine collected to serve as blank samples for method development and validation.

For biliary excretion study, bile duct of the rats was surgically cannulated under light ether anesthesia [4]. Rats were dosed with the solution formulation of CDRI-85/92 at 20 mg/kg orally or intravenously and placed in Bollman cages. Bile samples were collected in glass tubes at 0–12 and 12–24 h post dose. The volume of the samples were recorded and stored at −60°C until analyses.

### Partition coefficient (log P)

Determination of log P was carried out according to the method of Said et al. (1996) [5]. 1-Octanol and 20 mM phosphate buffer (pH, 7.4) were mutually saturated for 6 h on magnetic stirrer and separated. A solution of the compound (1 mg/ml) was prepared in octanol saturated buffer. Buffer (2 ml) and octanol containing CDRI-85/92 were placed in seven glass tubes and screw capped. These tubes were tumble-mixed for 2 h at 25°C at about 100 inversions/min. The organic and aqueous phases were separated by centrifugation at 2500 rev/min for 20 min. The concentration of the compound in aqueous and organic phase was determined by HPLC after a suitable dilution in mobile phase. The log P was calculated from the following equation:

$$\text{Log P}=\text{log}\frac{{\text{C}}_{\text{octanol}}}{{\text{C}}_{\text{buffer}}}$$

where C_octanol_ and C_buffer_ are the concentrations of the compound in octanol and buffer, respectively. Further, log P values {Log P= log10 (pKa)} were also calculated after conducting potentiometric titration. It was carried out manually using a pH electrode and a variable volume pipette. A solution of the compound (1 mg/ml) was prepared in water and it was titrated with a 0.1 M NaOH solution, pH was measured simultaneously. The equivalence point (pK_a_- partition coefficient) was estimated from the inflection point in the titration curve from the plot between pH and volume of mL of NaOH solution.

### Bioanalyses in Serum

Serum concentrations of the compound were determined by HPLC method as described by Srivastava & Gupta (2002) [6] with minor modification. Briefly, to 0.5 ml serum (blank, spiked or test) in 10 ml test tube 1.5 ml of acetonitrile was added and vortex-mixed for 15 sec. The tubes were kept in fridge for 30 min for complete protein precipitation, vortex-mixed for 1 min and centrifuged at 1000 × g for 10 min at 4°C. Supernatant (1.5 ml) was transferred to a 10 ml conical tube without disturbing the lower precipitate pellet and evaporated to dryness in Savant speed vac concentrator. The residue was reconstituted in 0.1 ml of mobile phase, centrifuged at 1000 × g for 10 min and the resulting solution was injected onto the HPLC system.

### Bioanalyses in Urine, bile and feces

The HPLC method described above was validated for estimation of CDRI-85/92 in urine and bile by assaying the spiked control samples in these biomatrics at three concentration levels (20, 50 and 100 ng/ml of the drug). To have a robust method, inter- and intra-day validation was also conducted (Table 2).

For the preparation of the spiked control feces samples, weighed (25 mg) quantities of drug-free powdered feces were transferred to different glass tubes, fortified with appropriated volume of working dilutions of CDRI-85/92 prepared in methanol, mixed by gentle tapping and allowed to stand at room temperature for drying. The blank, spiked or test samples were moistened with 0.2 ml of KH_2_PO_4_ buffer (20 mM, pH 3.0) followed by addition of 1.0 ml of acetonitrile for protein precipitation. The tubes were sealed with parafilm and allowed to stand at room temperature with frequent vortexing. Supernatant were processed as described above for serum samples. For each analytical run, a calibration curve was constructed using peak response against the concentration in calibration standards prepared in same biomatrix.

### Data analyses

After oral dose, serum peak concentration (C_max_) and the time to reach peak concentration (T_max_) were derived directly from the original data. The serum concentration-time data from intravenous and oral dosed rats were fitted to WinNonlin programme (version 1.5) to calculate various pharmacokinetic parameters (like AUC, Cl and Vd). The bioavailability for oral dose was calculated using the following equation:

$$\%\text{Bioavailability}=\frac{{\text{AUC}}_{\text{oral}}\times {\text{Dose}}_{\text{iv}}}{{\text{AUC}}_{\text{iv}}\times {\text{Dose}}_{\text{oral}}}\times 100$$

The amount, cumulative amount and % of the administered dose excreted via urine, bile and feces were calculated.

## Results and Discussion

Proton pump inhibitors (PPIs) are highly effective and well tolerated therapeutic agents, making them the drug of choice for the treatment of patients having increased gastric acid secretion [7]. Here, we present the result of pharmacokinetics and excretion studies of CDRI-85/92, a proton pump inhibitor, developed by our Institute.

The chromatographic conditions and the extraction procedure gave a clean chromatogram for the compound in the biometrics. The procedure of sample preparation was easy and required a simple protein precipitation by acetonitrile. Recovery from serum varied from 89 to 94% with coefficients of variation less than 5.6%. The moderate analysis time (10 min) together with rapid sample preparation allowed rapid analyses. The calibration curve was linear over the range 1.25–200 ng/ml. Using 0.5 ml serum, the lower limit of quantitation was 1.25 ng/ml after a five-fold concentration. The mean observed concentrations of the quality control samples deviated less than 10% from nominal values for the compound and the highest between and within-assay precision (coefficient of variation) was <15%. The HPLC method provided reproducible estimates of the compound in serum with sufficient sensitivity to allow pharmacokinetic and bioavailability studies. Here we have presented the intra- and inter-day accuracy and precision data for urine, feces and bile (Table 2).

The log P of the compound CDRI-85/92 was 0.53 ± 0.11 and 0.55 ± 0.18 by octanol partition and potentiometric titration, respectively. Being hydrophilic, it should be eliminated at a faster rate. Log P in the range of 0–3 [8] also points towards the suitability of the CDRI-85/92 for oral administration.

**Fig. 2:**
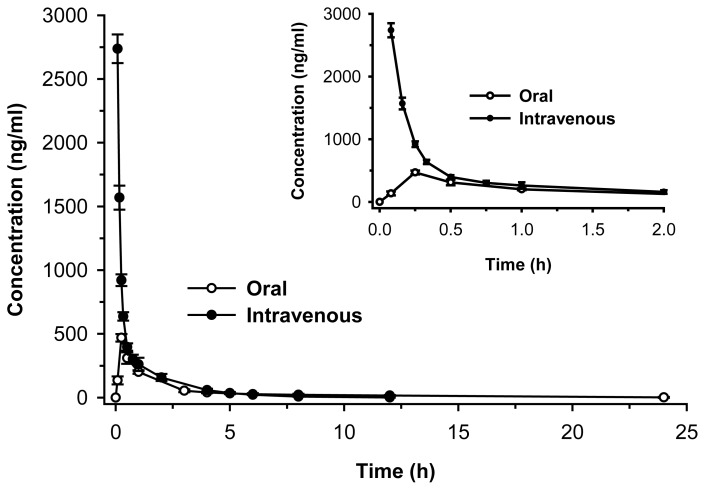
Concentration-time profile of CDRI-85/92 after 20 mg/kg oral and intravenous treatments

The bioavailability of CDRI-85/92 has not been reported to date. In this study, the pharmacokinetics was estimated in male *Sprague Dawley* rats after 20 mg/kg oral and intravenous dose. Since the compound was freely soluble in water we had preferred aqueous solution of CDRI-85/92 in iv and oral treatment. The serum concentration-time data for the compound after intravenous and oral administration is shown in Figure 2. After intravenous administration, CDRI-85/92 was detected in serum up to 12 h. The serum concentration-time data was best fitted to two-compartment open model with elimination from a central compartment (using WinNonlin). Table 3 summarizes the pharmacokinetic parameters calculated from the serum concentration-time data. CDRI-85/92 was eliminated with an elimination half-life of 2.1 h and may have a longer duration of acid inhibition. Large volume of distribution (greater than 0.7 L/kg in case of rats) indicated the availability of compound in the tissue to exert its prolonged action. A PPI with a longer half-life should induce more prolonged blockade of proton pumps and is hence likely to bring about greater suppression of gastric acid secretion [9]. Currently available benzimidazole-based PPIs have half-lives of 1–2 h in human [10, 11]. The half-life of omeprazole is less than 5 min in rats [12], in comparison CDRI-85/92 has a half-life of 2 hr in rats which can lead to superior and sustained control of acid secretion at night [10].

Following 20 mg/kg oral administration, the compound showed peak concentration (C_max_) of 469.2 ± 45.5 ng/ml after 1 h of dose (absorption phase). The compound appeared in the blood as early as 5 min post oral treatment with C_max_ occurring at 1 h post dose (Table 3). The AUC (882 ng.h/ml) was about half as compared to that after intravenous administration (1251 ng.h/ml). The systemic bioavailability (F) of the compound was 70.5% after oral administration. As a result of prolonged absorption, as indicated by an MAT of 2.8 h, the MRT (3.9 h) after oral administration was considerably longer than that observed after an intravenous administration (1.1 h). The pharmacokinetic data corroborates with the protein binding (32%) and pH independent absorption profiles of CDRI-85/92 [13]. Some of the oral pharmacokinetics studies of CDRI-85/92 and its ester prodrug have also been reported previously [14, 15]. However, they reflect only the comparison of the oral pharmacokinetics of CDRI-85/92 and its ester derivative in different formulation (not aqueous) since ester prodrug was not fairly water soluble. This might be the reason that an increased C_max_ and T_1/2_ was noticed based on the well known formulation enhancement effects.

**Fig. 3:**
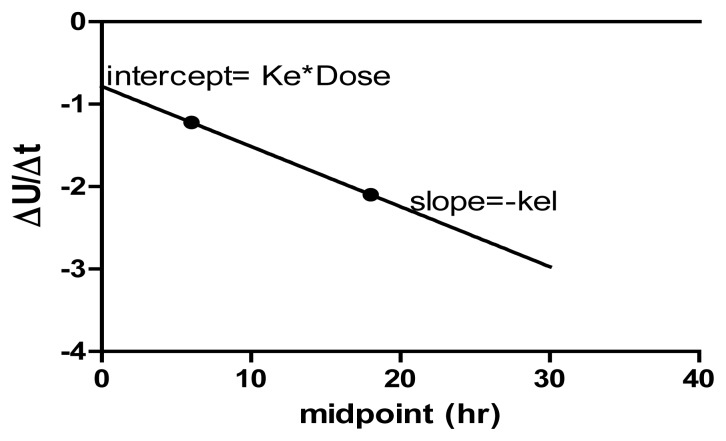
Representative (urine oral noncannulated) Semi-log Plot of ΔU/Δt versus Time midpoint, where K_el_ is elimination rate contant, K_e_ is excretion rate constant

Excretion of CDRI-85/92 was studied in rats at 20 mg/kg oral and intravenous dosage regimen. Urinary excretion of CDRI-85/92 was 4.2 ± 1.1 and 15.7 ± 3.3% of the oral and intravenously administered CDRI-85/92 dose, respectively (Table 4). Further, in bile-cannulated rats it was found to be 1.7 ± 0.8 and 16.8 ± 2.7%, respectively. In all the cases, the amount of the drug excreted was much more in the time range of 0–12 h as compared to 12–24 h. No difference was observed in the urinary excretion of CDRI-85/92 when comparison was made between cannulated and non-cannulated rats after intravenous treatment. However, bile duct cannulated rats showed difference in urinary excretion after oral dose. Fecal excretion of CDRI-85/92 up to 24 h amounted to 1.2 ± 0.6% and 0.08 ± 0.08% of the oral and intravenously administered CDRI-85/92 dose, respectively (Table 4). Biliary excretion of CDRI-85/92 (up to 24 h) after oral administration was found to be 0.37 ± 0.12% of the administrated dose whereas in case of intravenous treatment, the fecal and biliary levels of CDRI-85/92 were below the quantitation limits. Further, the excretion parameters (Table 5) ie. f_e_: fraction of drug excreted, K_el_: elimination rate contant, K_e_: excretion rate constant were calculated based on the semi log plot between ΔU/Δt versus Time_midpoint_ (Fig. 3). However, a disadvantage of this plot is that the error present in “real” data can obscure the straight line and lead to results which lack precision. Also, it was difficult to collect frequent, accurately timed urine samples. This is especially true when the elimination half-life is small, as is the case with our compound. The results thus negate the enterohepatic recycling of the drug, which corroborates with the single AUC and T_max_ values we obtained in the concentration-time profile. The excretion data clearly state that renal clearance is one of the major means for the excretion of CDRI-85/92 in urine. Further, a trans-isomer of CDRI-85/92 was detected in the urine samples after oral treatment of CDRI-85/92. The data obtained in the pharmacokinetics and excretion studies corroborates with the metabolism studies of the compound in which very slow metabolism was noticed by glutathione-S-transferases with the formation of trans-isoform [16]. This is also evident by low clearance of CDRI-85/92. This clearly points towards the hydrophilic nature of the compound and correlates with the log P values obtained.

In conclusion, the results of this study provide the complete analyses of pharmacokinetics and excretion of CDRI-85/92 in *Sprague Dawley* rats, one of the preclinical species used in toxicological studies. Pharmacokinetic parameters of CDRI-85/92, after oral and intravenous treatment, depict that the compound has an adequate bioavailability. Excretion studies reveal the involvement of renal clearance of the compound (15% of the administered dose) via urine.

## Figures and Tables

**Tab. 1:** Sampling Schedule during oral and iv treatment

Animal grouping													

	Time points (hr)												
	
Groups (n=3)	0.08	0.25	0.5	1	2	3	4	5	6	8	12	24	48
A													
B													
C													
D													

**Tab. 2:** Intra- and Inter-day accuracy and precision in urine, feces and bile samples

Nominal Concentrati on (ng/mL)	Observed Concentration (ng/mL)			Accuracy %			Precision (% RSD)		

	Urine	Feces	Bile	Urine	Feces	Bile	Urine	Feces	Bile

	**Intra Day**								

20	20.12	19.87	19.74	100.6	99.35	98.7	10.93	11.58	9.84
50	49.45	48.26	49.67	98.9	96.52	99.34	7.31	8.32	7.54
100	98.13	97.99	99.77	98.13	97.99	99.77	6.27	15.56	3.11

	**Inter Day**								

20	20.67	20.03	20.84	103.35	100.15	104.2	11.78	5.45	10.99
50	51.23	47.29	51.56	102.46	94.58	103.1	8.23	12.35	10.59
100	102.67	99.78	100.1	102.67	99.78	100.1	5.28	6.75	3.93

**Tab. 3:** Pharmacokinetic parameters of CDRI-85/92 after 20 mg/kg in rats

Parameters	IV Treatment	Oral Treatment
C_max_ (ng/ml)	4907.2	469.2
T_max_ (h)	–	1.00
Elimination t_1/2_ (h)	2.1	
AUC_0-∞_ (ng.h/ml)	1251	882
Cl (L/h)	1.1	
Vd (L/kg)	3.7	
MRT (h)	1.1	3.9
Bioavailability (%)	–	70.5
MAT (h)	–	2.8

C_max_, peak concentration; T_max_, time to C_max_; elimination t_1/2_, elimination half-life; AUC_0-∞,_ area under the concentration-time curve from zero to infinity; Cl, Clearance; Vd, volume of distribution; MRT, mean residence time; MAT (mean absorption time) = MRT_oral_ MRT_IV_.

**Tab. 4:** Excretion Profile of CDRI-85/92 dose in rat urine, feces and bile after 20 mg/kg oral and intravenous (iv) treatment

Treatment	% of dose recovered								

	Urine			Feces			Bile		

	0–12 h	12–24 h	Total	0–12 h	12–24 h	Total	0–12 h	12–24 h	Total

Oral (NC)	3.7	0.5	4.2	1.0	0.2	1.2	–	–	–
IV (NC)	15.7	ND	15.7	0.05	0.02	0.08	–	–	–
Oral (BC)	1.5	0.2	1.7	–	–	–	0.3	0.08	0.37
IV (BC)	16.8	ND	16.8	–	–	–	ND	ND	–

NC, non-cannulated; BC, bile duct cannulated; ND, not detected.

**Tab. 5:** Excretion Parameters of CDRI-85/92 after 20 mg/kg dose in rat urine, feces and bile after oral and intravenous (iv) treatment

Treatment	Excretion Parameters								

	Urine			Feces			Bile		

	f_e_	K_e_ (hr^−1^)	K_el_ (hr^−1^)	f_e_	K_e_ (hr^−1^)	K_el_ (hr^−1^)	f_e_	K_e_ (hr^−1^)	K_el_ (hr^−1^)

Oral (NC)	0.56	0.04	0.07	0.012	0.07	0.06	–	–	–
IV (NC)	0.9	0.09	0.1	0.0008	0.15	0.03	–	–	–
Oral (BC)	0.83	0.06	0.07	–	–	–	0.0037	0.1	0.048
IV (BC)	0.90	0.04	0.05	–	–	–	–	–	–

where f_e_ is fraction excreted, K_el_ is elimination rate contant, K_e_ is excretion rate constant.
